# Synthesis, In Vitro and In Silico Analysis of New Oleanolic Acid and Lupeol Derivatives against Leukemia Cell Lines: Involvement of the NF-κB Pathway †

**DOI:** 10.3390/ijms23126594

**Published:** 2022-06-13

**Authors:** Gianfranco Fontana, Natale Badalamenti, Maurizio Bruno, Davide Castiglione, Monica Notarbartolo, Paola Poma, Alberto Spinella, Marco Tutone, Manuela Labbozzetta

**Affiliations:** 1 Dipartimento di Scienze Biologiche, Chimiche e Farmaceutiche (STEBICEF), University of Palermo, Viale delle Scienze ed. 17, 90128 Palermo, Italy; natale.badalamenti@unipa.it (N.B.); maurizio.bruno@unipa.it (M.B.); monica.notarbartolo@unipa.it (M.N.); paola.poma@unipa.it (P.P.); manuela.labbozzetta@unipa.it (M.L.); 2 Dipartimento di Chimica, University of Turin, Via Pietro Giuria 7, 10125 Turin, Italy; davide.castiglione@unito.it; 3 Centro Grandi Apparecchiature (CGA)—ATeN Center, University of Palermo, Via F. Marini 14, 90128 Palermo, Italy; alberto.spinella@unipa.it

**Keywords:** Oleanolic acid, Lupeol, HL60, HL60R, antitumor activity, NF-κB, docking

## Abstract

Oleanolic acid (OA) and Lupeol (LU) belong to the class of natural triterpenes and are endowed with a wide range of biological activities, including cytotoxicity toward several cancer cell lines. In this context, we investigated a set of compounds obtained from the two natural precursors for the cytotoxicity against leukemia HL60 cells and the multidrug-resistant (MDR) variant HL60R. Six new semi-synthetic triterpenes have been synthetized, fully characterized, and were investigated together with other triterpenes compounds for their pharmacological mechanism of action. The interaction of the more cytotoxic compounds with the nuclear factor kappa B (NF-κB) pathway has been also evaluated with the aid of docking. The lupane-like compounds were more active than the precursor, while the oleane-like compounds showed more complex behavior. Both OA and LU derivatives possess a similar interaction pattern with the p65 subunit of NF-κB, justifying the similar trend in their ability to inhibit the binding of p65 to DNA. Further, some of the derivatives tested were able to increase IκB-α levels preventing the translocation of NF-κB to the nucleus. In conclusion, this study offers a deeper insight on the pharmacological action of triterpenes toward leukemia cells, and it improves the background useful for the development of new anti-cancer drugs.

## 1. Introduction

Cancer has been considered a major cause of death worldwide and despite substantial advances in diagnosis and treatment, current therapeutic protocols such as radiotherapy, chemotherapy, immunotherapy, and surgical protocols are limited and often ineffective, suggesting a crucial need to find new effective therapeutic agents. Although there have been significant achievements in the development of synthetic drugs, natural products still play an important role in drug discovery. Indeed, particularly in cancer therapy, about 80% of clinically used agents are inspired or directly derived from natural products [1]. In the last few years, there has been a growing interest in natural triterpenoids due to their versatile biological activities [2]. Several triterpenoids have shown promise as antineoplastic agents and exhibit anti-proliferative activity when tested against various cancer cell lines [3,4,5,6].

### 1.1. Oleanolic Acid and Lupeol

**Scheme 1:**
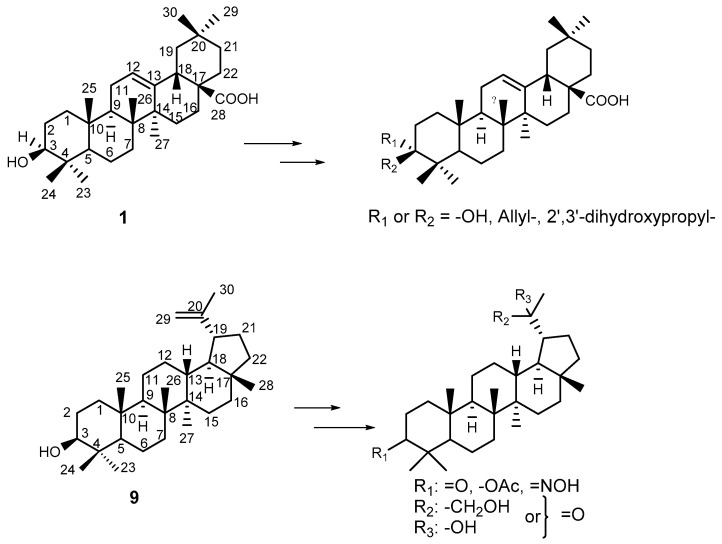
Structures, numbering and functional modifications of the starting natural compounds OA **1** and LU **9**.

Current studies on olive oil triterpenoids, such as Oleanolic acid (3β-hydroxyolean-12-en-28-oic acid, OA **1**, Scheme 1), have revealed a marked potential in altering different cell signaling pathways and thus reveal significant potential in cancer prevention and therapy [7,8,9,10,11]. OA is one of the most common pentacyclic triterpenoids compounds mainly found in different herbal sources [12]. In plants and plant-based foods, OA is often found together with its isomer Ursolic acid, from which it differs by the sites of the methyl group on the E loop. Both isomers have similar pharmacological properties [13,14], although they can differ in the intensity of biological activity due to the difference in the position of the methyl groups, which influences their potency and consequently bioactivity of compounds [15,16]. Many studies have shown that OA has liver protection, antioxidation, hypolipidemia, antitumor, anti-inflammatory, and antiviral pharmacological effects [17,18,19]. OA has been shown to act at various stages of tumor development inhibiting tumor initiation and promotion, as well as inducing tumor cell differentiation, apoptosis, inhibiting angiogenesis, invasion and metastasis [20]. Moreover, OA has been shown to have direct antitumor activity synergistically acting with chemotherapy drugs: it is able to inhibit efflux transporters, thus increasing the intracellular concentration of the chemotherapy drugs. Anticancer properties of OA are highlighted in the low micromolar range [20]. However, due to its poor solubility in water and the low bioavailability of oral administration, the development of OA in the pharmacological field has been limited and its therapeutic effect is also difficult to fully exert [21]. A promising solution is to modify the molecular structure of OA to obtain more bioactive derivatives, which could be the basis for the development of new drugs [22].

Lupeol (Lup-20(29)-en-3β-ol, LU **9**, Scheme 1) also known as Fagarsterol, is one of the better-explored plant-derived triterpenes found in lupines, white cabbage, green peppers, strawberries, olives, mangoes, and grapes. It was reported to possess beneficial effects as a therapeutic and preventive agent for a range of disorders, showing anti-inflammatory, cardioprotective, anti-diabetic, skin protective, gastroprotective, anti-microbial, antiproliferative, and antitumor activities [23,24,25,26,27,28,29]. Recently, studies have been carried out to investigate the structure-activity relationships of LU in various human cancer cell lines [30,31]. The mechanism of action of LU suggests that it is a multi-target agent. In particular, the anti-tumor promotion effects of LU were observed to be associated with its potential to modulate signaling pathways such as NF-κB and the phosphatidylinositol 3-kinase [PI3K]/Akt (protein kinase B pathway), which are reported to play an important role during tumorigenesis [32]. LU was shown to significantly inhibit the NF-κB translocation and its DNA binding activity in a mouse model of skin tumorigenesis [33]. Like other terpenoids contained in herbal medicine and natural products also, LU may inhibit efflux pumps, causing changes in drug bioavailability [34]. A study conducted by Aratanechemuge and colleagues showed that LU induces apoptosis of human promyelocytic HL60 leukemia cells [30]. It is known that LU at its effective therapeutic doses (40 mg/kg) [35] does not show toxicity to normal cells and tissues. It has also been reported that LU exhibits low acute systemic toxicity, its antiproliferative and cytotoxic effects on tumor cells occur at concentrations up to 50–800 µM [36], therefore LU derivatives with higher potency would be preferable.

### 1.2. Semi-Synthetic Derivatives of the Two Natural Triterpenoids Precursors

**Scheme 2:**
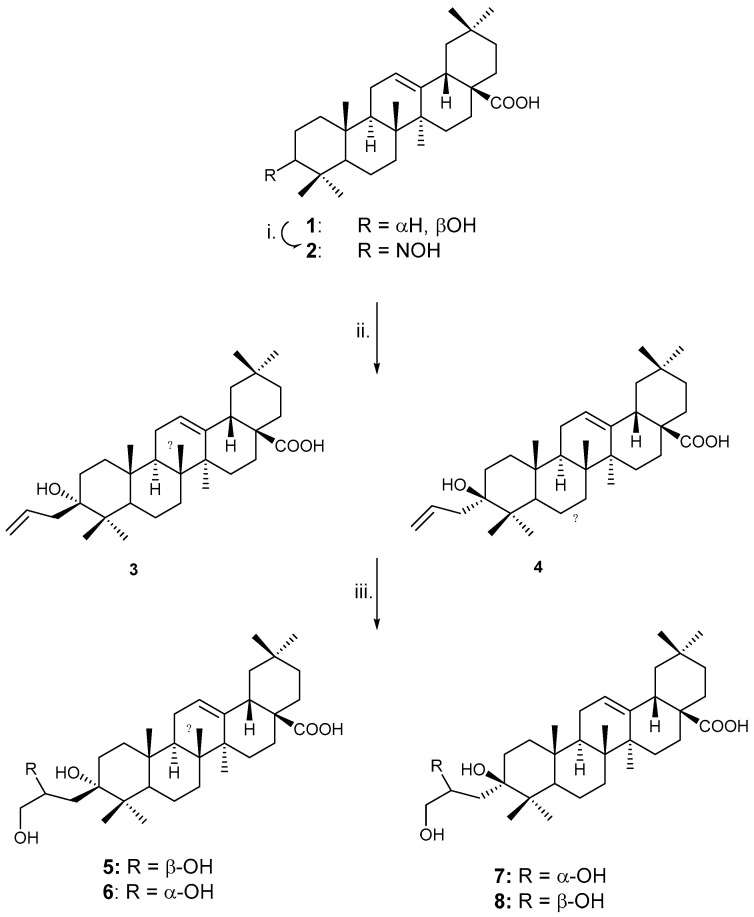
Preparation of compounds **2**–**8** from OA **1**: i. (1) Jones reagent, Me_2_CO, 0 °C, (2) NH_3_OHCl, Py, rt; ii. (1) Jones reagent, Me_2_CO, 0 °C, (2) AllylMgCl, dry THF, 0 °C; iii. OsO_4_ (cat.), NMO, rt, Me_2_CO/H_2_O6:1.

**Scheme 3:**
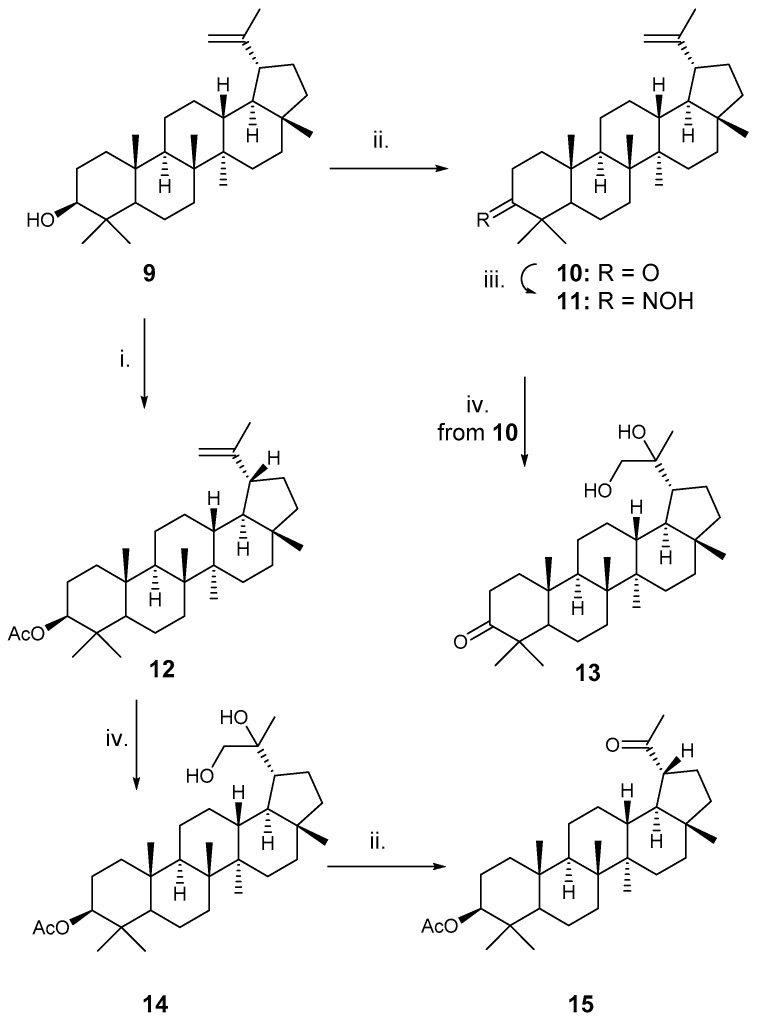
Preparation of compounds **10**–**15** from LU **9**: i. Ac_2_O/Py, rt; ii. PCC, 3 Å ms, dry DCM, rt; iii. NH_2_OH.HCl, 3 Å ms, dry Py; iv. OsO_4_ (cat.), NMO, Me_2_CO/H_2_O 6:1.

For all previously made assumptions, in this study, a set of derivatives of the two natural triterpenoids, OA and LU, were prepared and tested to verify their biological activities on acute promyelocytic leukemia (APL), a subtype of acute myeloid leukemia cancer cell HL60 and its MDR variant HL60R. The MDR variant cell line was obtained by us through the exposure of the sensitive cell line HL60 to increasing doses of doxorubicin. Treatment with doxorubicin modified the phenotype of the HL60 cell line by inducing the expression of several factors responsible for multidrug resistance. The HL60R cell line is characterized, in fact, by the overexpression of the P-glycoprotein efflux pump, constitutive activation of the transcription factor NF-κB, and as a consequence of the overexpression of inhibitor of apoptosis proteins (IAPs) [37]. A number of chemical transformations (Scheme 1, Scheme 2 and Scheme 3) of the two precursors were selected in order to modify their structure in regions that were demonstrated as particularly crucial for the interactions with the bio-environment. These are the C-3 in OA and the C-3, C-20, and C-29 in LU. In a previous study, we showed how the introduction of a new lipophilic three-carbon chain in place of H-3α of OA can determine the inhibition of NF-κB activation in hepatocellular carcinoma (HCC) cell lines overexpressing this factor and characterized by innate drug resistance [16]. Following these precedents, we investigated the effect of this modification also on the HL60 and HL60R cells and compared the eventually different effects associated with the presence of a hydrophilic chain in substitution of the hydrophobic one. As regards LU, we designed some modifications aimed to modify the H-donor/acceptor possibilities of the molecule at the above-mentioned positions by replacing the CH_2_-29 with oxygen or hydroxymethyl and the β-OH at C-3 with acetoxyl, carbonyl and hydroxyimino groups. These derivatives were assessed for their cytotoxicity and the mechanism of action was investigated by evaluating the interaction of the most active compounds with the NF-κB pathway in the MDR cell line. A deeper insight into the interaction pattern of both the classes of structures with the p65 subunit has been gained by a docking study.

## 2. Results and Discussion

### 2.1. Chemistry

The obtainment of compounds **3**–**8** from natural OA **1** is reported in Scheme 2, while the preparation of derivatives **10**–**15** from natural LU **9** is reported in Scheme 3. The already described [30] Jones oxidation at C-3 of compound **1** gave the corresponding ketone, the precursor of the hydroxyimino derivative **2**, obtained upon treatment with NH_2_OH.HCl in dry Pyridine. Unlike for **1**, the Jones reagent was unsuccessful for **9** probably because of the sensitivity shown by this compound toward the acidic conditions [38]. On the other hand, it was possible to convert **9** to the 3-oxo-derivative **10** by treatment with PCC in dry DCM. Compound **10** was subsequently converted to the oxime **11** by the standard treatment with hydroxylamine hydrochloride in Pyridine. The C-3 epimers **3** and **4** were prepared by allylation in Barbier-Grignard conditions following our previously reported procedure [16]. Compound **9** was acetylated with Ac_2_O/Py to give the 3-acetoxy-derivative **12**. The dihydroxylation procedure, involving the use of a catalytic amount of OsO_4_ combined with stoichiometric N-Methylmorpholine-N-oxide (NMO) let us convert compounds **3**, **4**, **10**, and **12** to the corresponding glycols: **5**–**8**, **13**, and **14**. Finally, unusual degradative oxidation of the glycol **14** promoted by PCC gave the nor-lupane derivative **15**. Both the ^1^H and the ^13^C NMR spectra (Table 1) of the new compounds **5**-**8** were characterized by the disappearance of the signals arising from the alkenyl moieties at C-2′ and C-3′ in **3** and **4**. On the other hand, new signals for oxygenated functions appeared in the spectra of the four compounds as doublets at 70.3, 70.3, 70.8, and 69.6 ppm and as triplets at 68.6, 68.8, 68.2, and 68.6 ppm. The determination of the absolute configuration at the new chiral center at C-2′ of **5**–**8** was rather arduous because of the lack of significant evidence obtainable from NOESY spectra. For this reason, a specific ab initio computational protocol has been developed, that will be illustrated in detail in a separate paper. In brief, the shielding tensors for the ^13^C resonances have been computed for all the possible absolute configurations at C-2′ at DFT-B97-2 level with a pcs-seg-2 basis set after DFT-B3LYP geometry optimization with Grimme’s correction for dispersion interactions. The theoretical values obtained were compared with the experimental chemical shifts and the results let us unambiguously assign the configurations as *S, R, S,* and *R* (Scheme 2) for the four compounds, respectively.

The chemical structures of the new lupane derivatives **13** and **14** were obtained by spectral analysis (Table 2), including 1D and 2D hetero-correlated NMR spectra. In particular, the two oxygenated carbons of the new alcoholic fragments appear as a singlet at ca. 75 ppm and a triplet at ca. 68 ppm for compounds **13** and **14**. The two protons at C-29 give their signals as doublets at ca. 3.40 and 3.60 ppm in both the compounds.

### 2.2. Cytotoxic Activity on HL60 and HL60R Cell Lines

The cytotoxic effects of different compounds on cell lines were evaluated by MTS assay after 72 h of treatment. The concentrations of the drug which caused 50% inhibition of cell growth (IC_50_) are reported in Table 3 and in the Supplementary Materials (Figure S1–S4).

Cell growth inhibition assays revealed that the cytotoxic activity of most compounds on the HL60 cell line and on its MDR variant HL60R cell line is quite equivalent. All of the derivatives of OA, excluding **6**, were more potent than the original compound; the derivatives **5**, **7**, and **8** show comparable IC_50_ values; **3** and **4** have IC_50_ lower than half compared to OA **1**. Interestingly, compound **2** shows significantly lower IC_50_ compared to OA even if different between the two lines (4.5 µM in HL60 cell line and 11.2 µM in MDR cell line). Further, comparing the values obtained for compounds **3**–**8**, it can be inferred that the stereochemistry at C-3 has no significant effect on the cytotoxicity (**3** and **5** vs. **4**, **7**, and **8**), while the stereochemistry at C-2′ is quite important in the C-3α epimers as the activity strongly falls down on passing from the *S* to the *R* configuration at C-2′ (**5** vs. **6**). The results obtained for LU derivatives show that all derivatives are significantly more potent than the precursor **9**, which has an IC_50_ > 100 µM. Further, the anti-proliferative and cytotoxic effects on tumor cells, differently from those reported in the literature [32], occur at concentrations lower than 50 µM. Again, most of the compounds showed comparable activity in the two cell lines. Only derivatives **14** and **15** show different activity between the two lines and have an IC_50_ of 9.6 µM and 21.2 µM in the HL60 cell line and 14.5 µM and 43.2 µM in the MDR cell line, respectively, while the compound **10** behaves like LU **9**.

### 2.3. Effects of Derivatives on NF-κB (p65 Subunit) Pathway in HL60R Cells

**Figure 1:**
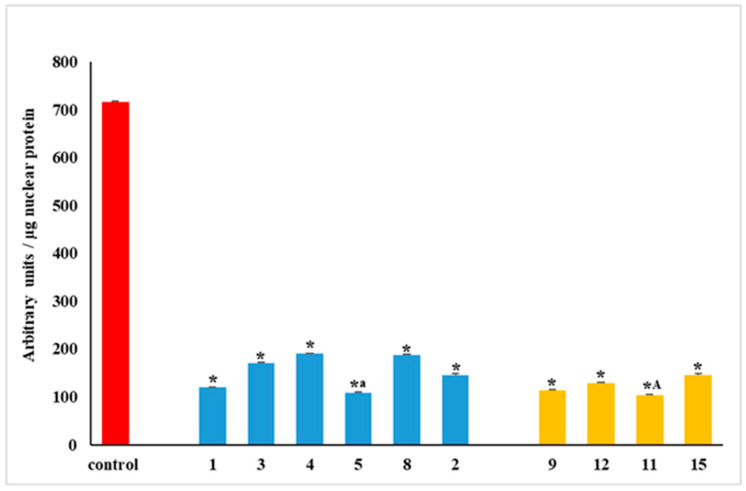
NF-κB (p65 subunit) DNA binding capacity in nuclear extracts of HL60R cell line. The cells were treated for 24 h with compounds at the corresponding IC_50_ values. Results are expressed as arbitrary units/μg protein of HL60R nuclear extracts and the mean ± SE of three independent observations, * *p* < 0.01 versus control; ^a^ *p* < 0.05: AO derivates versus precursor, ^A^ *p* < 0.05: LU derivates versus precursor (one-way ANOVA followed by Tukey’s test).

Several data in the literature reveal that the antitumor capacity of both triterpenoids, OA and LU, is associated with their potential to modulate the NF-κB signaling pathway [11,32,33]. Our results previously published indicated that the HL60 cells showed a very slight DNA binding capacity of the p65 subunit. Otherwise, the HL60R cells showed remarkable levels of the activated p65 subunit [37]. In order to verify the ability of the investigated compounds to interfere with the NF-κB DNA binding capacity, we analyzed the effects of the different derivatives on NF-κB activation by TransAM assay in the MDR cell line. For the analysis, derivatives with an IC_50_ overlapping in the two cell lines and which are more active than the original compounds were chosen. HL60R cells were treated with the derivatives at the corresponding IC_50_ values for 24 h. The results showed that, even if there is no direct correlation with the different toxicity of the compounds, all the derivatives considered resulted in a significant decrease in the binding capacity of NF-κB to the corresponding DNA consensus sequence (Figure 1).

**Figure 2:**
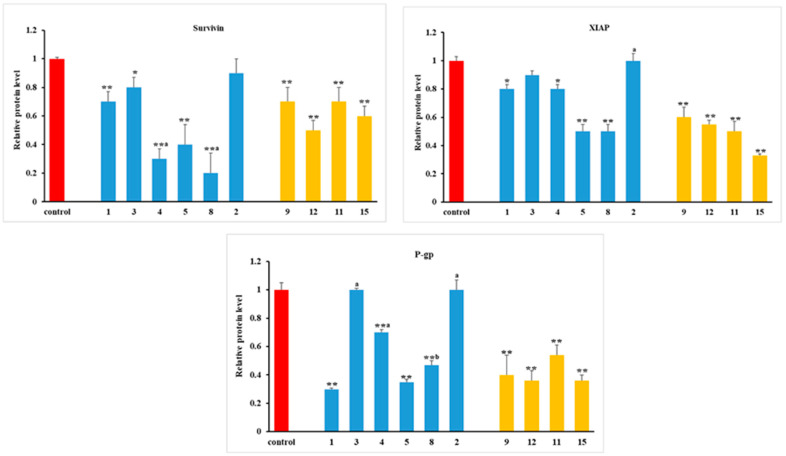
Western blotting analysis of the levels of Survivin, XIAP, and P-gp in HL60R cells. The cells were treated for 24 h with different compounds. Relative protein level (mean ± standard error of three experiments). Differences when treatments are compared to the control: ** *p* < 0.01, * *p* < 0.05; derivates versus precursors: ^a^ *p* < 0.05 (Survivin and XIAP), ^a^ *p* < 0.001 and ^b^ *p* < 0.05 (P-gp) (one-way ANOVA followed by Tukey’s test).

Given these results, we investigated if the treatment with these derivates at the same conditions also modified the expression of some NF-κB targets in the MDR variant cell line. The results obtained show inhibition of two important anti-apoptotic proteins, Survivin and XIAP, and of the efflux pump, P-gp. Specifically, LU and its derivatives seem to have a homogeneous action between them. On the other hand, different behavior is observed between the derivatives of OA, which does not correspond to their cytotoxic and NF-κB inhibitory capacity. For example, compounds **2** and **3** did not determine any modulation of these factors’ target of NF-κB (Figure 2). This data could be partially explained by the fact that other transcriptional factors can contribute to the regulation of these proteins. However, the reduction of P-gp expression confirms the important role of triterpenes in modulating the efflux system responsible for drug resistance and suggests their potential use as sensitizing agents (Figure 2).

### 2.4. Molecular Docking Analysis

**Figure 3:**
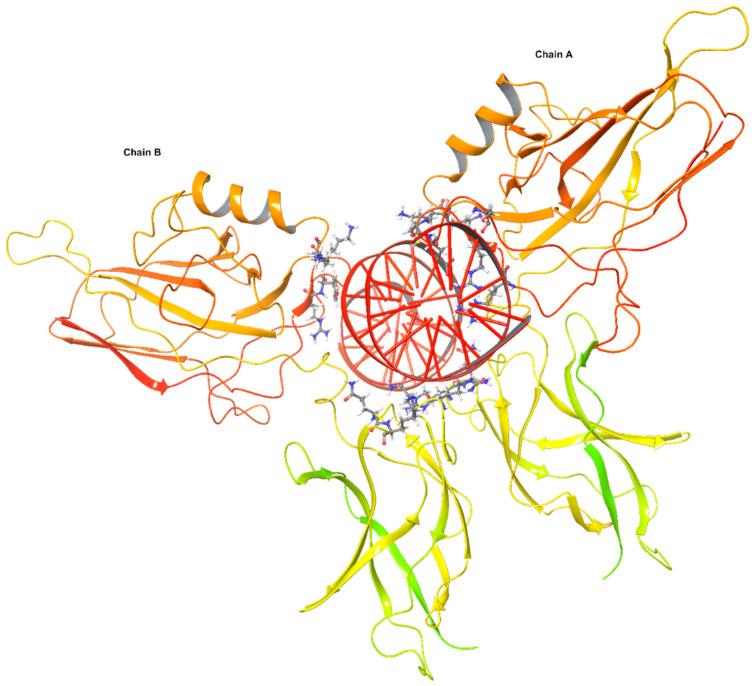
The crystal structure of the NF-kB p65 (PDB ID 2RAM) homodimer in complex with DNA. In colored ribbons the p65 subunits, in the red ribbon the DNA helix, in sticks are reported the residues that form H-bonds with the DNA helix.

With the aim of better understanding the molecular mechanism of p65 inhibition, docking studies were carried out for the compounds that most inhibited the NF-κB DNA binding capacity **1, 5**, **9**, and **11** with the X-ray crystal structure of the murine NF-κB p65/p65 homodimer in complex with a 20-mer DNA target (PDB ID: 2RAM) [39]. This structure was selected due to the high degree of amino acid sequence identity (>90%) between mouse and human p65 subunits. Unlike p50, the two p65 subunits are not symmetrically disposed on the DNA target. The local dyad axis of the protein dimerization domains is not coincident with the DNA dyad. The homodimer should optimally bind to a pseudo-palindromic nine base pair target with each subunit recognizing a 5′GGAA-3′ half-site separated by a central A-T base pair. However, subunit B is oriented differently from its counterpart. The N-terminal domain of subunit B is rotated 18 degrees from its normal conformation. Remarkably, subunit B retains all the interactions with the sugar-phosphate backbone of the DNA target. This mode of interaction allows the NF-κB p65 homodimer to recognize DNA targets containing only one cognate half-site (Figure 3).

These variations in conformation on different κB targets may add an additional level of gene regulation by this protein family. For these reasons, we decided to perform the docking analysis on both subunits. The grid of docking was centered on the residues that form H-Bonds with the DNA dyad (Figure 3). The p65 subunit A establishes H-bonds with Arg33, Arg35, Cys38, Glu39, Ser42, Lys122, Lys123, Arg246, and Gln247, while the grid of docking for the p65 subunit B was centered on Arg35, Tyr36, Lys122, Lys123, Gln220, Lys221, Arg246, and Arg247. Both identified binding pockets are characterized by two lips of positively charged residues that can host the negative phosphates of the double-stranded DNA and a deep pocket formed by neutral residues.

**Figure 4:**
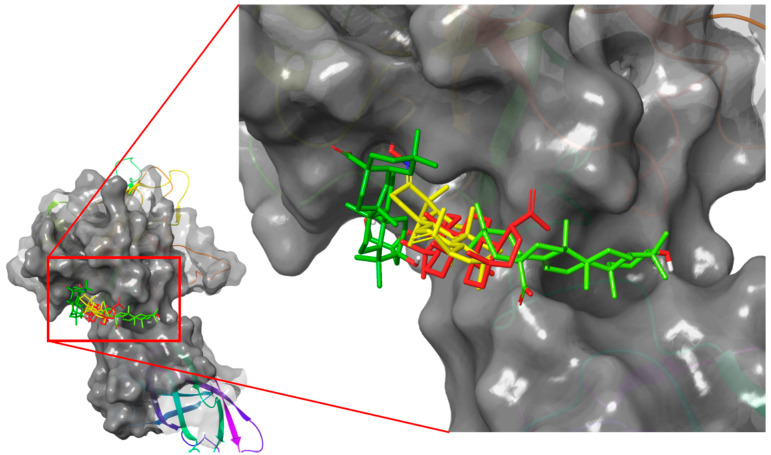
Best docking poses of the compounds **1** (light green), **5** (dark green), **9** (red), and **11** (yellow) in the chain A of the p65 (PDB ID: 2RAM).

**Figure 5:**
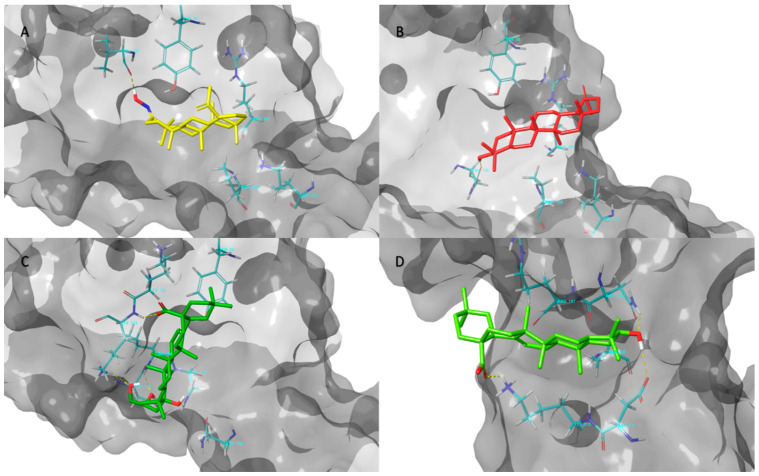
Ligands interactions with the p65 chain A residues. The yellow dashed lines represent the H-bonds interactions, and the light blue residues represent positive vdW contacts with the ligands. **11** (**A**), **9** (**B**), **5** (**C**) and **1** (**D**).

All the compounds analyzed i.e., **1**, **5**, **9**, and **11** showed higher binding energy with the p65 subunit A with respect to the p65 subunit B. In terms of residues involved in the binding of the compounds, there is a similar involvement in both subunits even though the grids have been centered in a different fashion. For these reasons, we will discuss the best pose of each active compound in terms of binding energy. Docking of compound **11**, the most active compound, to the p65 subunit A revealed a binding mode characterized by the lupane framework oriented in a parallel fashion to the nucleotide T11 of the co-crystallized DNA strand chain C. The cyclopentyl ring of the lupane framework is directed outwards with respect to the binding pocket, and the ring A extends towards the deep bottom of the binding pocket, forming one H-bond with the hydrogen atom of the oxime group in position 3 of the ring A and the carbonyl oxygen atom of the Val121. The binding mode of compound **11** is settled by vdW contacts with Tyr36, Arg187, Pro189, and Lys218 by the three central rings of the lupane framework. The other LU derivative which showed the higher capacity to bind the NF-κB p65 subunit was compound **9**. Upon docking **9** into the subunit A, we obtained a complex showing an overall binding mode similar to **11** (Figure 4 and Figure 5), with the ligand presenting the lupane framework oriented in a parallel fashion to the nucleotide T11 with the cyclopentyl ring pointing in the same direction as **11**, but this time the allyl group in position 19 of the pentacyclic ring of the lupane framework is oriented inwards facing the residue Arg33 and Arg187. Ring A, similarly to the previous one, extends towards the deep bottom of the binding pocket but interacts with the amide oxygen atom of the residue Asn155 through the hydrogen atom of the hydroxyl moiety in position 3. To confirm the high similarity in the binding mode of the two LU derivatives, Tyr36, Arg187, Pro189, and Lys218 form favorable vdW contacts with the ligand, too. The other two compounds that showed the higher capability to bind the NF-κB p65 subunit are two oleanane framework derivatives, OA, and compound **5** characterized by the substitution of one hydrogen atom at C-3 with a 2,3-dihidroxypropyl moiety. The lupane and the oleanane framework derivative give different binding modes. In fact, the docking of **5** showed an almost orthogonal orientation of the five-fused ring system with respect to the lupane framework.

The five fused ring system of **5** occupies the top side of the binding pocket characterized by positively charged residues such as Lys122 and Lys123, that are responsible for the interaction with the phosphate groups of the nucleotide chains. Both types of derivatives point their hydroxyl groups into the deep pockets. The primary hydroxyl of the propyl moiety forms an H-bond with the ε-NH_2_ group of Lys123. The same residue establishes an H-bond with the α-amino group and the carboxyl moiety of the molecule. Compound **5** forms a third H-bond with its hydroxyl bound to the five fused rings and the amide nitrogen of the Asn155, and at the same time, the secondary hydroxyl establishes two H-bonds with Asp153 and Leu154. Positive vdW contacts realized with Tyr36, Ly122, Lys123, Tyr152, Asp153, Leu154, Asn155, and Asn190 enhance the ligand-target interaction. Docking of OA in the NF-κB p65 subunit showed a unique binding mode with respect to the previous compounds. The compound occupies the top of the binding pocket interacting with a series of different residues. In particular, it establishes three H-bonds, the first with its carbonyl oxygen and the epsilon amino group of the Lys218. The other two H-bonds are related to the interaction of the hydroxyl hydrogen at C-3 and the γ acid group of Asp217 and the amino group of Asn186. Additionally, for this compound, positive vdW contacts with Asn186, Arg187, Ala 192, Asp217, and Lys218 support the right fit in the binding pocket. Overall, docking evidence confirms that both the OA and LU derivatives possess a similar dynamic of interaction with the p65 unit of NF-κB that justifies the similar trend in their ability to inhibit the binding of p65 to DNA, regardless of the different effects on cell viability.

### 2.5. Effects of Derivatives on IκB-α Protein Expression

**Figure 6:**
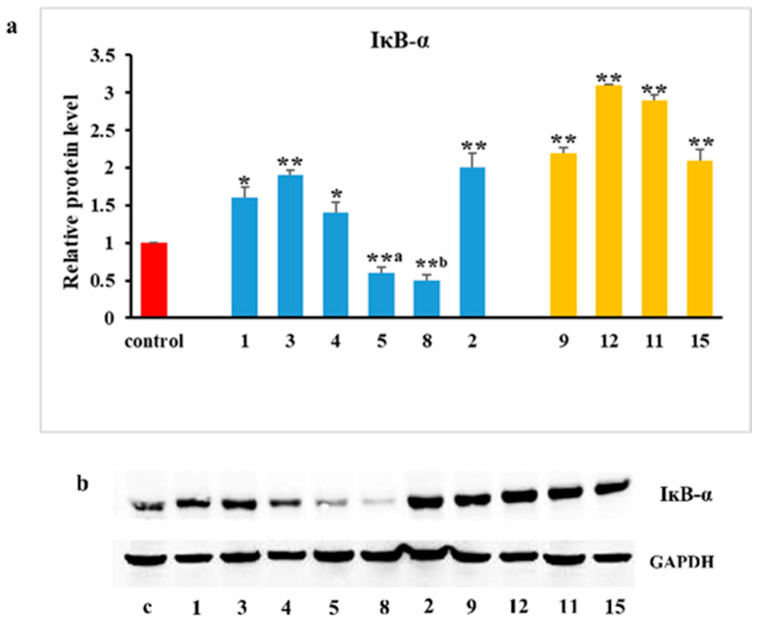
Western blotting analysis of the levels of IĸB-α in HL60R cells. The cells were treated for 24 h with different compounds. (**a**): Relative protein level (mean ± standard error of three experiments). Differences when treatments are compared to the control: ** *p* < 0.01, * *p* < 0.05; derivates versus precursor: ^a^ *p* < 0.05; ^b^ *p* < 0.01 (one-way ANOVA followed by Tukey’s test). (**b**): Representative immunoblots of three independent experiments with similar results.

The NF-κB transcription factors are present in the cytosol in an inactive state complexed with the inhibitory IκB proteins [40]. Activation occurs via phosphorylation of IκB-α at Ser32 and Ser36 followed by proteasome-mediated degradation that results in the release and nuclear translocation of active NF-κB [41,42,43]. In order to further explore the biomolecular mechanism of p65 inhibition, we also investigated if the treatment with the OA and LU derivatives at the same conditions also modified the expression of the IκB-α factor. All of the termini of the LU series (**9**, **11**, **12**, **15**) were able to induce an increase in the IκB-α factor (Figure 6).

On the other hand, the effect observed for the AO series is more complex: some compounds show a positive effect compared to the control, i.e., **2** and **3** (*vide supra*) and to a lesser extent **1** and **4**, while other compounds show an opposite effect **5** and **8**. Following these results, it seems likely that the most active compounds within the Olean series may act in different stages of the NF-κB pathway, i.e., **5** prevents the binding of p65 to DNA, while **2** prevents translocation to the nucleus. LU derivatives, on the other hand, seem to have a similar spectrum of activity, all probably acting with a double mechanism against NF-κB. It is likely that many of the compounds are able to increase IκB-α levels, preventing the transcription factor from translocating to the nucleus and thus explaining an indirect action of inhibition of the factor and some of its targets. Indeed, other reported data show how some AO derivatives can directly block the activity of IKK-β and therefore the NF-κB pathway in human myeloid leukemia cells (U937) by interacting with cysteine (Cys179) in the cycle activation of IKK-β [44]. It is also known that radicals such as hydrogen peroxide (H_2_O_2_) affect the degradation of IκB-α through tyrosine phosphorylation [45], therefore it is possible to hypothesize that these triterpene substances, possessing a known antiradical action [46,47,48], reduce ROS levels causing an increase in IκB-α and consequently a reduction in NF-κB activation.

## 3. Materials and Methods

### 3.1. General

Reaction progress was monitored by TLC on silica gel plates (Merck 60, F254, 0.2 mm). Organic solutions were dried over Na_2_SO_4_. Evaporation refers to the removal of solvent on a rotary evaporator under reduced pressure. The NMR spectra were recorded on a Bruker Avance II 400 apparatus operating at 400.15 and 100.63 MHz for ^1^H and ^13^C, respectively, at the temperature of 300 °K. The instrument was equipped with an inverse broadband probe (BBI). For the acquisition of 1H spectra, an 11.87 μs 90° pulse, a delay time of 5 s, and 16 scans were used. ^13^C spectra were acquired using a 90° pulse of 12.2 μs, a decoupling pulse of 80 μs, and a delay time of 3 s. The 2D ROESY spectra were acquired at the same temperature using a spin-lock pulse of 300 ms, eight scans and 256 experiments. The 2D HSQC correlation spectra were acquired at the same temperature through a double inept transfer pulse sequence; the decoupling intervals were set as follows: a 90° pulse of 11.87 μs for ^1^H, with eight scans and 256 experiments. The 2D HMBC long-range correlation spectra were acquired through a J-filtered pulse sequence in order to suppress the single bond correlations with no decoupling during acquisition, using a 90° pulse of 11.87 μs on the ^1^H nucleus, eight scans and 256 experiments. The stationary phase for flash chromatography (FC) was silica gel Merck (Kiesegel 60/230–400 mesh). Optical rotations were determined on a Jasco P-1010 digital polarimeter. Microanalysis data (C, H) were obtained by an Elemental Vario EI. III apparatus and are consistent with ±0.4% of the theoretical value. Dry THF was obtained by standard distillation of commercial THF onto Na. All chemical reagents were purchased from Merck.

Compounds **2**–**4**, **10**–**12**, and **15** were recognized by comparing physical and spectral properties with literature data.

### 3.2. Preparation of Compound ***2*** from OA 1

An amount of 100 mg (0.213 mmol) of compound **1** was solubilized in 10 mL of Me_2_CO and the Jones reagent was introduced dropwise at 0 °C until a pale yellow color developed in the solution. The green solid residue was filtered off and the solvent was removed by the rotavapor. The residue was purified by FC (light petroleum/acetone 5 to 10% in acetone) to give 3-oxooolean-12-en-28-oic acid in quantitative yield. The product was recognized by comparison with an authentic sample available from previous work [30]. Then, 50 mg (0.106 mmol) of the obtained compound was treated with 1.5 eqs. (0.159 mmol, 11 mg) of Hydroxylammonium chloride in 3 mL of dry Pyridine and the mixture was stirred overnight. Then, the solvent was removed by co-evaporation with toluene and the residue was portioned between water (5 mL) and CHCl_3_ (5 mL). The water phase was extracted with CHCl_3_ (3 X 5 mL) and the combined organic phase was dried onto Na_2_SO_4_ and distilled to remove the solvent. The residue was purified by FC (cyclohexane/EtOAc 10:1) to give 32 mg (62%) of 3-hydroxyiminoolean-12-en-28-oic acid (**2**) [49].

### 3.3. Preparation of Compounds ***3*** and ***4*** from 1

An amount of 500 mg (1.10 mmol) of compound **1** was oxidized as described above to give 3-oxooolean-12-en-28-oic acid that was treated with an excess of allyl magnesium chloride solution (2.5 mL of a 2 M solution) in 5 mL of dry THF, via syringe under Ar atmosphere at a temperature of 0 °C. The mixture was left to warm until rt overnight and then was poured into an iced 1 M HCl solution, adjusting the pH to 2. Then, the mixture was extracted with CHCl_3_ (4 × 30 mL), and the organic phase was washed with water and dried over anhydrous Na_2_SO_4_. The solvent was distilled under a vacuum and the residue was purified by FC (cyclohexane/acetone 9:1) to give compounds **3** (81 mg, 15% from **1**) and **4** (250 mg, 46% from **1**), identical in all respects to the authentic samples available from previous work [16].

### 3.4. General Procedure for the Dihydroxylation of Compounds ***3***, ***4***, ***10***, and ***12*** to Give ***5***–***8***, ***13***, and ***14***

An amount of 0.5 mmol of the starting compound and 2.0 mmol (241.5 mg) of 97% NMO were solubilized in 15 mL of Me_2_CO/THF/H_2_O 6:7:1. Then, 0.3 equivalents of OsO_4_ (1.5 mL of a 2.5% *w*/*w* solution in 2-methyl-2-propanol) were added in one portion and the final solution was stirred at rt for 72 h. The reaction was quenched by adding 2 mL of saturated Na_2_S_2_O_3_and stirring the mixture for 10′. The organic material was extracted with CHCl_3_/EtOH 2:1 (3 × 20 mL) and the organic phase was dried and distilled. The residual was purified by LC employing the mobile phases described below.

(3β)-2′*S*,3-dihydroxypropyl-(3α)-hydroxyolean-12-en-28-oic acid (**5**): amorphous white solid, anal. C 74.60%, H 10.37%; calcd for C33H54O5, C 74.67%, H 10.25%. IR (ν_max_ cm^−1^): 3381 (OH), 3174 (OH), 1680 (COOH); ^1^H and ^13^C NMR: see Table 1.

(3β)-2′R,3-dihydroxypropyl-(3α)-hydroxyolean-12-en-28-oic acid (**6**): amorphous white solid, anal. C 74.57%, H 10.40%; calcd for C33H54O5, C 74.67%, H 10.25%. IR (ν_max_ cm^−1^): 3370 (OH), 3170 (OH), 1689 (COOH); ^1^H and ^13^C NMR: see Table 1.

(3α)-2′R,3-dihydroxypropyl-(3β)-hydroxyolean-12-en-28-oic acid (**7**): amorphous white solid, anal. C 74.53%, H 10.45%; calcd for C33H54O5, C 74.67%, H 10.25%. IR (ν_max_ cm^−1^): 3375 (OH), 3168 (OH), 1693 (COOH); ^1^H and ^13^C NMR: see Table 1.

(3α)-2′S,3-dihydroxypropyl-(3β)-hydroxyolean-12-en-28-oic acid (**8**): amorphous white solid, anal. C 74.55%, H 10.43%; calcd for C33H54O5, C 74.67%, H 10.25%. IR (ν_max_ cm^−1^): 3380 (OH), 3170 (OH), 1690 (COOH); ^1^H and ^13^C NMR: see Table 1.

20,29-dihydroxy-Lupan-3-one (**13**): amorphous solid, anal. C 78.60%, H 10.94%; calcd for C30H53O3, C 78.55%, H 10.99%. IR (ν_max_ cm^−1^): 3505, 3480, 1715, 1260, 1005.); ^1^H and ^13^C NMR: see Table 2.

20,29-dihydroxy-Lupan-3-ol acetate (**14**): amorphous solid, anal. C 76.41%, H 10.85%; calcd for C32H54O4, C 76.45%, H 10.83%. IR (ν_max_ cm^−1^): 3500, 3472, 1700, 1258, 1000.); ^1^H and ^13^C NMR: see Table 2.

### 3.5. Preparation of Compound ***11*** from ***10***

An amount of 50 mg (0.118 mmol) of compound **10** was treated with 1.5 eqs. (0.176 mmol, 12 mg) of Hydroxylammonium chloride in 3 mL of dry Pyridine and the mixture was stirred overnight. Then, the solvent was removed by co-evaporation with toluene and the residue was portioned between water (5 mL) and CHCl_3_ (5 mL). The water phase was extracted with CHCl_3_ (3 × 5 mL) and the combined organic phase was dried onto Na_2_SO_4_ and distilled to remove the solvent. The residue was purified by FC (cyclohexane/EtOAc 19:1) to give 35 mg (68%) of 3-hydroxyiminolup-20(29)-ene (**11**) [50]:

### 3.6. Preparation of Compound ***12*** from ***9***

An amount of 100 mg of compound **9** was solubilized in 3 mL of Ac_2_O/Py 1:2 and stirred overnight at rt. Then, the volatiles were removed by co-evaporation with toluene and the residue was purified by FC (cyclohexane/EtOAc 9:1) to give Lupeol acetate **12** [31] in quantitative yield.

### 3.7. Preparation of Compound ***15*** from ***14***

An amount of 60 mg (0.12 mmol) of compound **14** was mixed with 52 mg (0.24 mmol) of PCC in 2.5 mL of dry DCM. The solution was stirred under Ar for 3 h. Then, the mixture was percolated on a celite pad and the filter was washed with 15 mL of EtOAc/Cyclohexane 2:1. The solvent was removed by rotavapor and the solid residue was purified by FC (EtOAc/Cyclohexane 1:6) to give 46 mg (87%) of **15** [51].

### 3.8. Cell Cultures

The HL60 cells were obtained from ATCC (CCL-240, Rockville, MD, USA), while its variant HL60R was cultured as previously described [19]. HL60 and HL60R cells were cultured in Roswell Park Memorial Institute (RPMI) 1640 (HyClone Europe Ltd., Cramlington, UK) supplemented with 10% heat-inactivated fetal calf serum, 2 mM L-glutamine, 100 units/mL penicillin, and 100 µg/mL streptomycin (all reagents were from HyClone Europe) in a humidified atmosphere at 37 °C in 5% CO_2_.

### 3.9. Cell Growth Assays

The cells were seeded at 5 × 10^3^ cells/well onto 96-well plates and then incubated overnight. At time zero, the medium was replaced with a fresh complete medium, and agents were added in different concentrations. After 72 h of treatment, 16 μL of a commercial solution (obtained from Promega Corporation, Madison, WI, USA) containing 3- (4,5-dimethylthiazol-2-yl)-5-(3-carboxymethoxyphenyl)-2-(4-sulphophenyl)-2H-tetrazolium (MTS) and phenazine ethosulfate were added. The plates were incubated for 2 h in a humidified atmosphere at 37 °C in 5% CO_2_. The bioreduction of the MTS dye was assessed by measuring the absorbance of each well at 490 nm. Cell growth inhibition was expressed as a percentage of the absorbance measured in the control cells.

### 3.10. NF-κB Activation

The DNA-binding capacity of NF-κB (p65 subunit) was measured in the nuclear extracts of HL60R cells treated using the TransAM™ NF-κB and Nuclear Extract™ Kits (Active Motif, Carlsbad, CA, USA) according to the manufacturer’s instructions. Briefly, the determination is based on a 96-well plate to which an oligonucleotide containing the NF-κB consensus binding site (5′-GGGACT TTCC-3′) has been immobilized. The activated NF-κB contained in extracts specifically binds to this nucleotide. By using an antibody that is directed against an epitope on p65 that is accessible only when NF-κB is bound to its target DNA, the NF-κB bound to the oligonucleotide is detected. The addition of a secondary antibody conjugated to horseradish peroxidase provides a sensitive colorimetric readout that is quantified by densitometry. The specificity of the assay is confirmed by contemporaneous incubations in presence of an excess of the non-immobilized consensus oligonucleotide, as a competitor, or of a mutated consensus oligonucleotide. The results were expressed as arbitrary units: one unit is the DNA binding capacity shown by 2.5 μg of whole-cell extract from Jurkat cells stimulated with 12-O-Tetradecanoylphorbol-13-acetate (TPA) and calcium ionophore (CI)/μg protein of nuclear extracts.

### 3.11. Western Blotting

Whole-cell lysates were obtained from HL60R cells using radioimmunoprecipitation assay buffer (Santa Cruz Biotechnology Inc., Dallas, TX, USA), and 25 µg protein was subjected to 10% SDS-PAGE and transferred to nitrocellulose membrane (Amersham, Pharmacia Biotech, Milan, Italy) using a semi-dry fast blot apparatus (Bio-Rad, Milan, Italy). Membranes were blocked with 5% (*w*/*v*) BSA in PBS–0.1% (*v*/*v*) Tween 20 for 1 h and then filters were incubated with primary antibodies raised against GAPDH (1:20.000; Sigma-Aldrich Srl, Milan, Italy), XIAP (1:500; Cell Signaling Technology, Inc., Danvers, MA, USA), Survivin (1:2000, Abcam Limited, Cambridge, UK), P-gp (1:100, Invitrogen, Milan, Italy) and IĸB-α (1:1000; Cell Signaling Technology, Inc. Danvers, MA, USA). Hybridization was visualized using an enhanced chemiluminescence detection kit (SuperSignal West Femto Maximum Sensitivity Substrate, Thermo Scientific Life Technologies Italia, Monza, Italy) and the Versa DOC imaging system (BioRad Laboratories, Milan, Italy). Immunoblots were quantified by densitometry, normalized against GAPDH value, and expressed as relative protein levels.

### 3.12. Protein-Ligands Preparation, and Docking

Initial coordinates of the compounds were constructed by using ACD/Chemsketch 2019.2.1. The structures were energy-minimized and prepared using Schrödinger LigPrep v. 2018–4. The force field adopted was OPLS3e and Epik [52] was selected as an ionization tool at pH 7.2 ± 0.2. Tautomers generation was unflagged and the maximum number of conformers generated was set at 32, and the chiralities have been determined from the original 3D structures.

Since the in vitro experiments were performed on NF-κB p65 subunits, for the in silico experiments we utilized the NF-κB murine structure, deposited in the Protein Data Bank (PDB) [39] under PDB ID 2RAM in its homodimeric form composed of p65/p65 subunits in complex with a DNA target to 2.4 Å resolution for the search of druggable cavities on the NF-κB surface. All water molecules, DNA, and cofactors were removed. The PDB file was refined using the protein preparation wizard tool of Maestro Suite Software [53]. This tool allowed the protein structure optimization, including missing loops, side chains, and hydrogens, optimization of the protonation state in a pH range of 7.0 ± 2.0, and analysis of atomic clashes. PROPKA was used to check for the protonation state of ionizable protein groups. Arginine and lysine side chains were considered cationic in the guanidinium and ammonium groups, and the aspartic and glutamic residues were considered as anionic in the carboxylate groups. The protonation and flip states of the imidazole rings of the histidine residues were adjusted together with the side-chain amides of glutamine and asparagine residues in a process of H-bonding network optimization. Protein was refined using restrained minimization with OPLS3e as a force field.

Molecular docking was performed by using Glide [54] in extra-precision (XP) with the OPLS3e force field using no constraints. Van der Waals radii were set at 0.8 and the partial cutoff was 0.15 and flexible ligand sampling. Bias sampling torsion penalization for amides with nonplanar conformation and Epik state penalties were added to the docking score. The grid boxes were centered on p65 residues which establish H-bonds with the co-crystallized DNA. In order to check for binding pose convergence, the top ten poses were included within the docking output.

Prime/MM-GBSA was used for the estimation of ΔG_binding_. The MM-GBSA approach employs molecular mechanics, the generalized Born model, and the solvent accessibility method to elicit free energies from structural information circumventing the computational complexity of free-energy simulations wherein the net free energy is treated as a sum of a comprehensive set of individual energy components, each with a physical basis [55]. The conformational entropy change—TΔS—can be computed by normal-mode analysis on docking poses, but many authors have reported that the lack of the evaluation of the entropy is not critical for calculating the MM-GBSA (or MM-PBSA) free energies for similar systems [56,57,58,59,60]. For these reasons, the entropy term–TΔS was not calculated to reduce computational time. In our study, the VSGB solvation model was chosen using OPLS3e force field with a minimized sampling method.

### 3.13. Statistical Analysis

Results are given as means ± standard error (SE). Statistical analysis was carried out by analysis of variance (one-way ANOVA) followed by Tukey’s test. Statistica ver. 12 (StatSoft Inc, Tulsa, OK, USA. 1984–2014) was used as software for the analyses.

## 4. Conclusions

In the present study, we focused on the cytotoxic effect of different derivatives of OA and LU in acute myeloid leukemia cancer cell line HL60 and its multidrug-resistant (MDR) variant HL60R. The MDR cell line is characterized by a poor prognosis, multidrug resistance, constitutive expression of the transcription factor NF-κB, overexpression of P-gp, and inhibitor of apoptosis proteins (IAPs). The results show that most of the compounds were found to be more active than the original compound and the cytotoxic activity on the HL60 cell line and on its MDR variant HL60R cell line is quite equivalent. Furthermore, the results obtained show that both the derivatives of OA and LU exhibit the remarkable property to disturb the NF-κB molecular pathway both upstream and downstream to its activation in this MDR cell line. All the compounds possess the ability to inhibit the transactivation of the transcription factor in the HL60R cell line. Moreover, the docking analysis confirmed that both OA and LU derivatives possess similar interaction patterns with the p65 unit of NF-κB, justifying the similar trend in their ability to inhibit the binding of p65 to DNA and some of its targets. In addition, it is likely that the ability of many of the derivatives to increase IκB-α levels prevents the transcription factor from translocating to the nucleus and thus explaining an indirect action of inhibition of the factor. The results obtained allow us to hypothesize that pretreatment with these compounds could make cells more sensitive to standard chemotherapy drugs and could represent new possible therapeutic approaches for AML.

## Figures and Tables

**Table 1:** ^1^H and ^13^C NMR spectral data ^1^ of the new compounds **5**–**8**.

C/H	5		6		7		8	
1	35.6 t	1.22 m 1.34 m	35.6 t	0.94 m 1.34 m	33.5 t	1.10 m 1.35 m	34.9 t	1.22 m 1.40 m
2	33.8 t	1.57 m 1.76 m	33.8 t	1.20 m 1.73 m	28.2 t	1.99 m 2.05 m	33.8 t	1.54 m 1.76 m
3	77.6 s		76.6 s		77.0 s (78.7) *		77.3 s	
4	41.8 s		41.8 s		42.0 s		42.6 s	
5	51.3 d	1.38 m	51.7 d	1.40 m	53.5 d (54.5) *	1.08 m	54.6 d	1.08 m
6	21.3 t	1.41 m 1.50 m	19.9 t	1.41 m 1.47 m	20.00 t	1.27 m 1.40 m	20.3 t	1.45 m 1.50 m
7	31.6 t	1.30 m 1.32 m	31.6 t	1.29 m 1.37 m	33.3 t	0.83 m 0.90 m	31.6 t	1.20 m 1.42 m
8	37.9 s		37.8 s		37.6 s		38.1 s	
9	47.6 d	1.76 m	47.7 d	1.73 m	46.8 d	1.60 m	47.7 d	1.70 m
10	37.9 s		37.9 s		37.7 s		38.3 s	
11	24.1 t	1.92 m 1.93 m	24.4 t	1.89 m 1.91 m	23.8 t	1.80 m 1.96 m	24.5 t	1.91 m 1.95 m
12	123.5 d	5.28 t (3.15)	123.7 d	5.26 t (3.3)	122.4 d	5.39 t (3.2)	123.6 d	5.27 t (3.1)
13	145.1 s		145.2 s		144.9 s		145.2 s	
14	40.5 s		40.8 s		37.8 s		40.6 s	
15	28.8 t	1.10 m 1.81 m	28.8 t	1.10 m 1.78 m	31.01 t	1.24 m 1.70 m	29.0 t	1.08 m 1.82 m
16	24.4 t	1.61 m 2.03 m	24.5 t	1.59 m 2.02 m	26.1 t	0.99 m 1.00 m	24.1 t	1.64 m 2.04 m
17	43.0 s		43.0 s		42.2 s		42.9 s	
18	42.7 d	2.88 dd (13.9, 4.4)	42.7 d	2.86 dd (14.0, 4.5)	39.8 d	3.20 dd (13.6, 4.5)	42.8 d	2.87 dd (13.7, 4.7)
19	47.2 t	1.14 m 1.70 m	47.3 t	1.12 m 1.67 m	46.7 t	1.21 m 1.69 m	47.3 t	1.16 m 1.71 m
20	29.5 s		29.9 s		28.7 s		30.8 s	
21	34.9 t	1.22 m 1.34 m	34.9 t	0.95 m 1.33 m	34.9 t	1.08 m 1.33 m	34.7 t	1.22 m 1.35 m
22	34.1 t	1.56 m 1.77 m	34.1 t	1.55 m 1.75 m	34.8 t	1.03 m 1.24 m	34.2 t	1.55 m 1.77 m
23	17.7 q	0.84 s	17.7 q	0.83 s	23.8 q	1.01 s	24.6 q	0.90 s
24	19.9 q	0.82	24.0 q	0.91 s	19.4 q	1.05 s	20.0 q	0.92 s
25	15.0 q	0.97 s	15.5 q	0.92 s	16.1 q	0.85 s	16.2 q	1.02 s
26	17.8 q	0.96 s	17.8 q	0.83 s	17.5 q	0.92 s	17.9 q	0.86 s
27	26.5 q	1.22 s	26.4 q	1.19 s	24.7 q	1.10 s	26.1 q	1.22 s
28	181.8 s		181.9 s		nd		181.8 s	
29	33.5 q	0.94 s	33.6 q	0.95 s	31.0 q	0.85 s	33.2 q	0.95 s
30	24.0 q	0.97 s	24.0 q	0.95 s	23.8 q	0.92 s	24.0 q	0.98 s
1′	38.0 t	1.47 m 1.78 m	40.5 t	1.42 m 1.84 m	34.8 t	1.93 m 2.29 m	37.8 t	1.62 m 1.92 m
2′	70.3 d	4.04 d quint. (1.8, 5.4)	69.6 d	3.94 m	70.8 d	4.42 quint. (5.3)	70.3 d	3.92 quint. (5.7)
3′	67.8 t	3.49 dd (5.7, 10.6) 3.44 dd (5.6, 10.6)	68.6 t	3.48 dd (5.1, 10.9) 3.44 dd (6.4, 10.8)	68.2 t	3.93 dd (5.8, 10.6) 3.83 dd (5.11, 10.7)	68.6 t	3.49 dd (11.7, 6.8) 3.58 dd (11.6, 6.8)

^1^ Data collected in MeOH-d_4_ for compounds **5**, **6**, and **8**, in Py-d_5_ for **7**. * Data in parentheses are the values obtained for compound **7** in MeOH-d_4_, where it is sparingly soluble. Hence, the complete characterization of **7** was made in Py and a decoupled ^13^C NMR spectrum was registered in methanol in order to compare the signals necessary for the stereochemical analysis.

**Table 2:** ^1^H and ^13^C NMR spectral data of the new compounds **13** and **14** in CDCl_3_.

C/H	13		14	
1	39.5 t	1.91 m 1.41 m	38.4 t	1.70 m 1.00 m
2	34.1 t	2.40 ddd (7.8, 4.6, 4.6) 2.50 ddd (7.8, 5.0, 5.1)	24.8 t	1.60 m 1.63 m
3	218.4 s	-	81.0 d	4.48 dd
4	47.2 s	-	37.6 s	-
5	54.7 d	1.32 m	55.3 d	0.78 m
6	19.6 t	1.45 m 0.80 m	18.9 t	1.45 m 1.50 m
7	33.8 t	1.43 m 1.44 m	34.5 t	1.48 m 1.39 m
8	41.2 s	-	41.4 s	-
9	49.6 d	1.34 m	50.2 d	1.28 m
10	36.7 s	-	37.8 s	-
11	21.9 t	1.33 m 1.46 m	21.4 t	1.48 m 1.34 m
12	27.5 t	1.02 m 1.89 m	27.5 t	1.00 m 1.51 m
13	40.3 d	1.42 m	37.0 t	0.99 m 1.68 m
14	43.5 s	-	43.5 s	-
15	27.5 t	1.51 m 1.74 m	27.5 t	1.70 m 1.51 m
16	35.4 t	1.48 m 1.34 m	35.5 t	1.39 m 1.40 m
17	44.6 s	-	44.7 s	-
18	47.9 d	1.40 m	48.0 d	1.40 m
19	47.3 d	1.93 m	47.3 d	1.91 m
20	75.2 s	-	75.2 s	-
21	28.6 t	1.05 m 1.52 m	27.9 t	1.51 m 1.04 m
22	37.7 t	1.73 m 1.75 m	40.4 t	1.10 m 1.32 m
23	26.6 q	1.06 s	28.6 q	0.85 s
24	21.0 q	1.02 s	16.5 q	0.84 s
25	16.0 q	0.94 s	16.2 q	0.87 s
26	16.0 q	1.08 s	16.2 q	1.05 s
27	14.7 q	0.92 s	14.8 q	0.94 s
28	18.9 q	0.80 s	18.2 q	0.80 s
29	67.2 t	3.44 d (10.3) 3.65 d (10.3)	67.3 t	3.44 d (10.3) 3.63 d (10.3)
30	24.8 q	1.20 s	23.7 q	1.20 s
-COCH_3_	-	-	171.0 s	-
-COCH_3_	-	-	21.3 q	2.03 s

The two protons at C-29 give their signals as doublets at ca. 3.40 and 3.60 ppm in both the compounds. The osmylation of **12** was completely stereoselective, giving rise to the new compound **14** as a single epimer. Similarly, compound **13** formed with a very high stereoselectivity (67% de, as established by ^1^H NMR) as an inseparable mixture of epimers. The absolute stereochemistry of the major epimers of both **13** and **14** has not been ascertained.

**Table 3:** IC_50_ values of cell lines treated with compounds.

	HL60 IC_50_ (Mean ± SE)	HL60R IC_50_ (Mean ± SE)
**1**	44.0 ± 0.7	43.5 ± 10.3
**2**	4.5 ± 3.3	11.2 ± 3.9
**3**	20.2 ± 0.5	19.0 ± 0.3
**4**	12.7 ± 0.9	16.0 ± 0.7
**5**	35.7 ± 0.9	32.2 ± 1.2
**6**	72.2 ± 2.3	72.7 ± 3.4
**7**	33.0 ± 3.5	39.5 ± 3.2
**8**	32.0 ± 1.8	32.0 ± 1.1
**9**	>100	>100
**10**	>100	>100
**11**	18.2 ± 2.3	22.5 ± 8.1
**12**	25.5 ± 5.3	24.2 ± 5 8
**13**	36.7 ± 1.9	44.7 ± 0.9
**14**	21.2 ± 4.1	43.2 ± 3.4
**15**	9.6 ± 0.1	14.5 ± 4.9

All the IC_50_ values are expressed in µM and the mean ± SE is calculated out of three independent observations.

## Data Availability

Not applicable.

## Supplementary Materials

The following are available online at https://www.mdpi.com/article/10.3390/ijms23126594/s1.
