# Supplementary figures and images for: Synthesis, In Vitro and In Silico Analysis of New Oleanolic Acid and Lupeol Derivatives against Leukemia Cell Lines: Involvement of the NF-κB Pathway
Source: Int J Mol Sci. 2022 Jun 13;23(12):6594. doi: 10.3390/ijms23126594 (PMC9223357; doi:10.3390/ijms23126594)

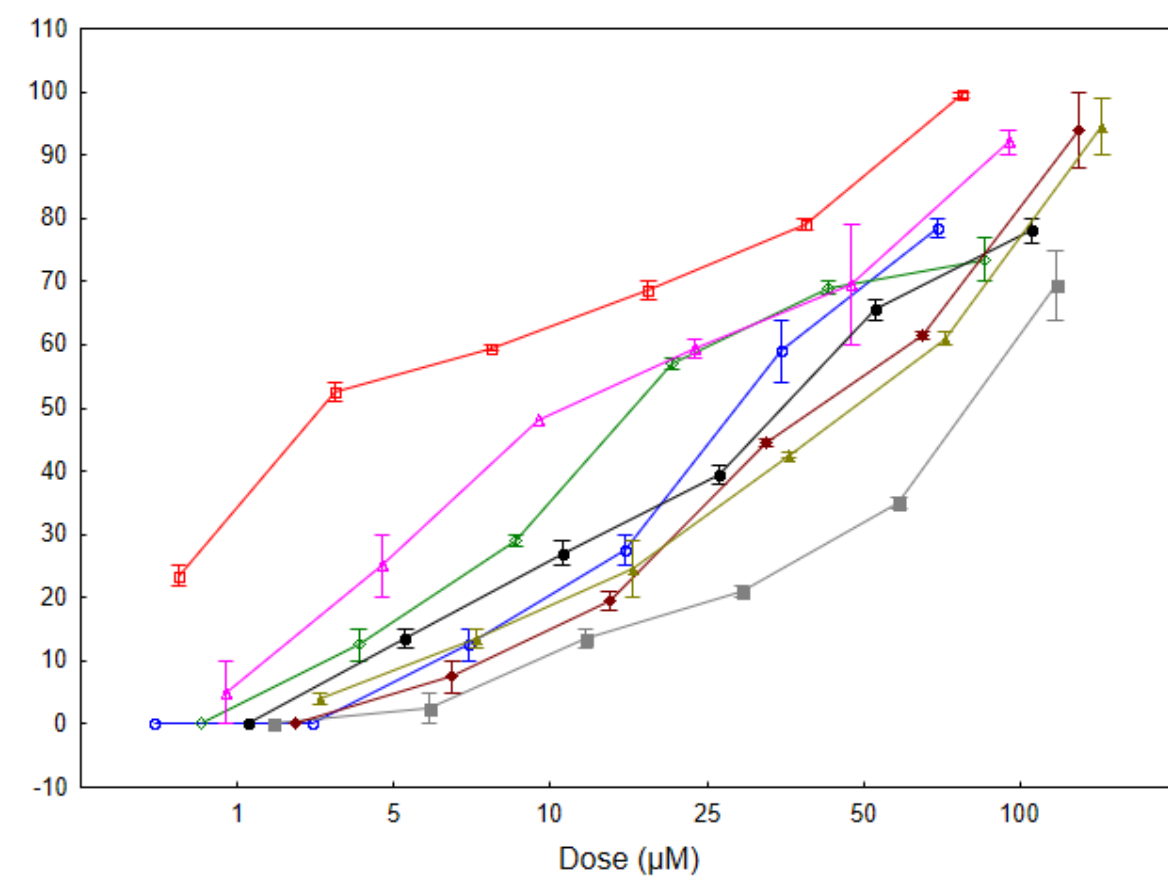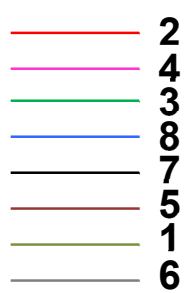

**Figure S1. HL60 cells**

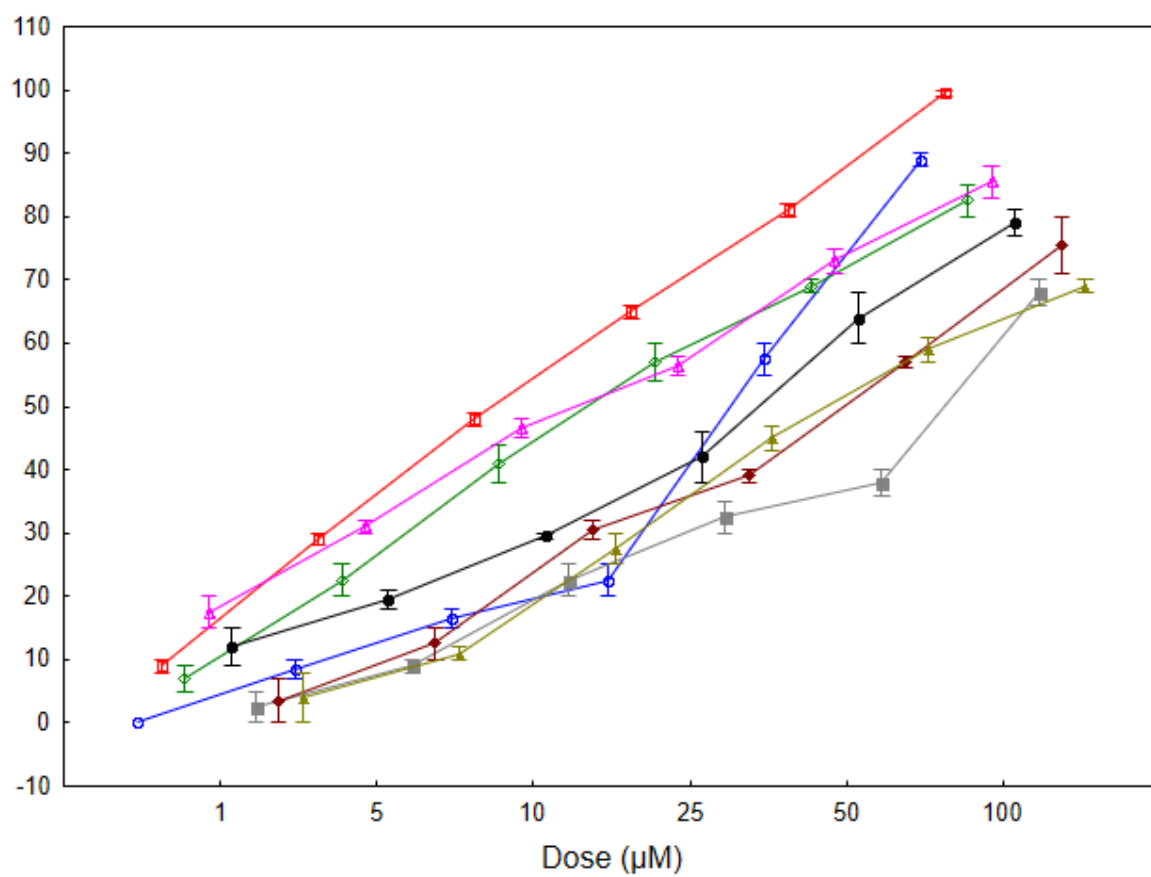

— 2  
 — 4  
 — 3  
 — 1  
 — 5  
 — 7  
 — 8  
 — 6

**Figure S2.** HL60R cells

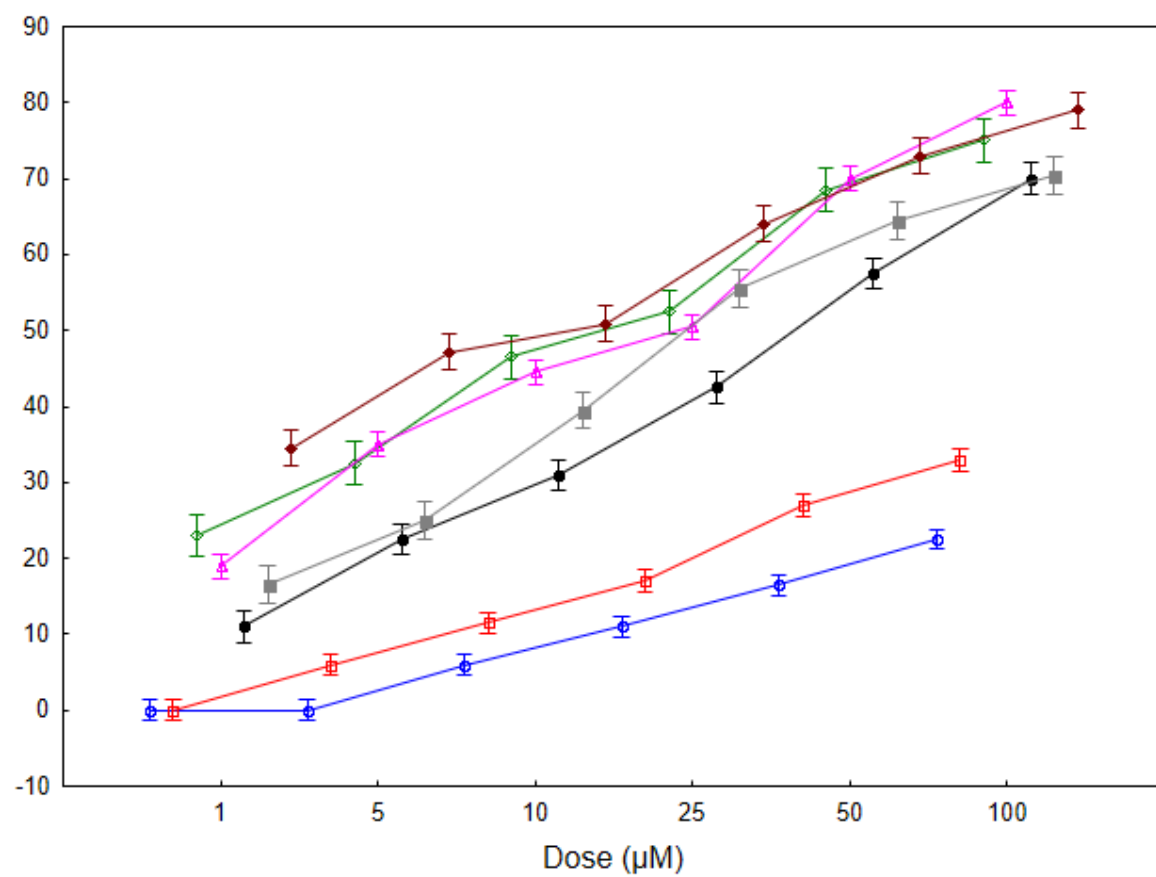

— 10  
 — 14  
 — 11  
 — 9  
 — 13  
 — 15  
 — 12

**Figure S3.** HL60 cells

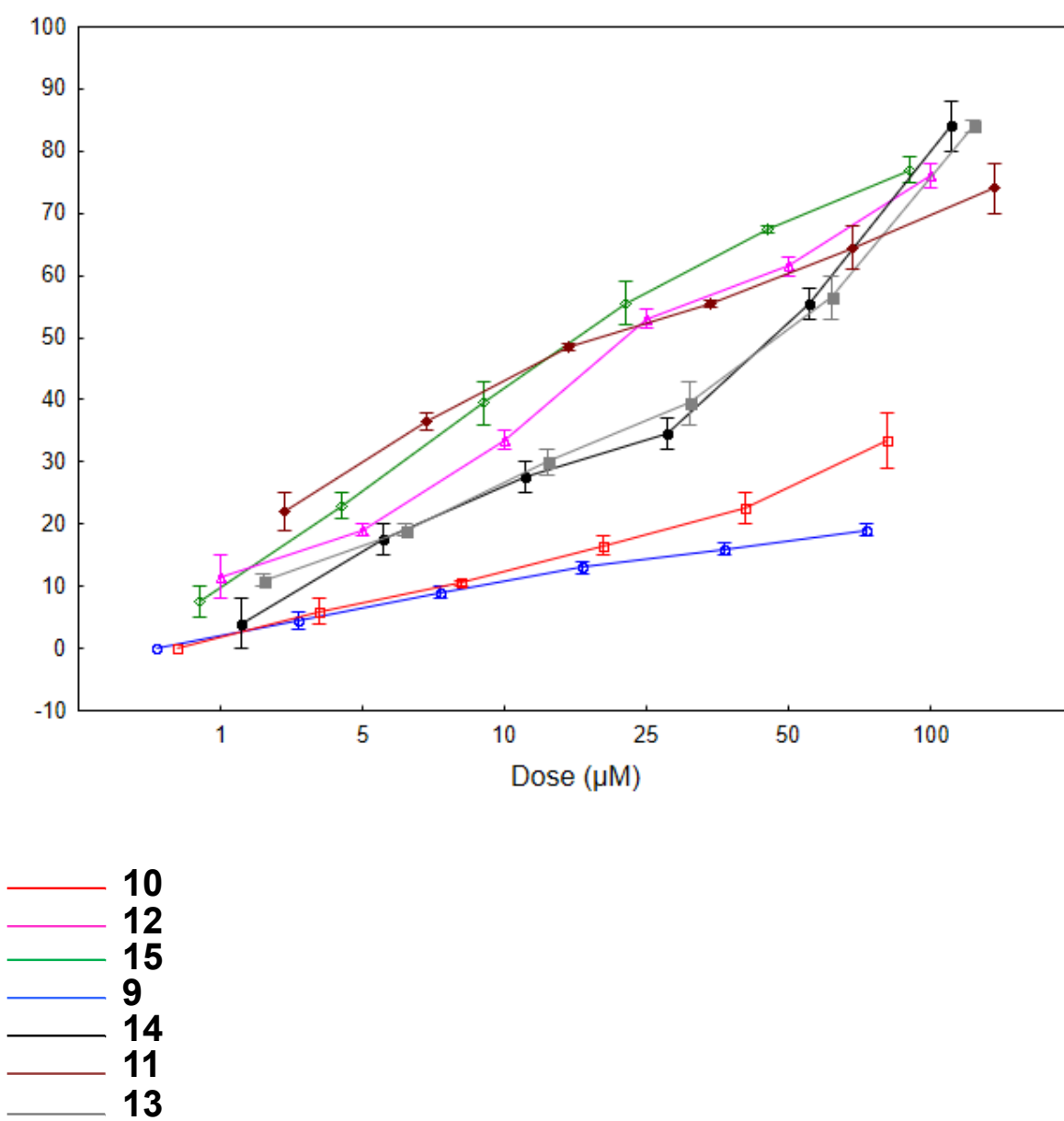

**Figure S4.** HL60R cells

Supplement: Supplementary file 1 [file ijms-23-06594-s001.zip › ijms-1751344-supplementary.pdf]
